# Supplementary material for: NAD+/NADH redox alterations reconfigure metabolism and rejuvenate senescent human mesenchymal stem cells in vitro
Source: Commun Biol. 2020 Dec 15;3:774. doi: 10.1038/s42003-020-01514-y (PMC7738682; doi:10.1038/s42003-020-01514-y)
Supplement: Supplementary file 2 — Supplementary Information [file 42003_2020_1514_MOESM2_ESM.pdf]

Supplementary information

NAD+/NADH Redox Alterations Reconfigure Metabolism and Rejuvenate Senescent Human Mesenchymal Stem Cells *In Vitro*

Xuegang Yuan[1, 2, *], Yijun Liu[1, #], Brent M Bijonowski[1, #], Ang-Chen Tsai[1, #], Qin Fu[1, #], Timothy M. Logan[3], Teng Ma[1], Yan Li[1, *]

1. Department of Chemical and Biomedical Engineering, FAMU-FSU College of Engineering, Florida State University, Tallahassee, FL, USA
2. Center for Interdisciplinary MR, National High Magnetic Field Laboratory, Tallahassee, FL, USA
3. Department of Chemistry and Biochemistry, Florida State University, Tallahassee, FL, USA

Supplementary Methods

Supplementary Method M1. Osteogenic and adipogenic differentiation of hMSCs.

Osteogenic differentiation: hMSCs were grown to confluence before CCM was switched to osteogenic differentiation medium containing high glucose DMEM (Gibco, Grand Island, NY), 10% FBS, 1% penicillin/streptomycin, 100 mM dexamethasone, 10 mM sodium-β-glycerophosphate, and 0.05 mM ascorbic acid-2-phosphate. The media were changed every 2 days and the differentiation was maintained for 14 days. Cells were then collected for RT-PCR. The differentiation can be visualized by Von Kossa staining under microscope [1].

Adipogenic differentiation: hMSCs were grown to confluence before CCM was switched to adipogenic differentiation medium containing high glucose DMEM, 10% FBS, 1% penicillin/streptomycin, 0.2 mM indomethacin, 0.5 mM isobutyl-1-methyl xanthine, 1 μM dexamethasone, 10 μg/mL insulin, and 44 mM sodium bicarbonate. Medium was changed every 2 days and the differentiation was maintained for 14 days. Cells were then collected for RT-PCR. The differentiation can be visualized by Oil Red O staining under microscope [1].

Supplementary Method M2. Measurement of indoleamine 2,3-dioxygenase (IDO) activity.

For IDO enzymatic activity, including both IDO1 and IDO2 (both convert Tryptophan to Kynurenine) was assessed by measuring Kynurenine level in cell culture supernatant. A 400 μl supernatant from hMSCs culture (either stimulated by IFN-γ at 40 ng/mL or left unstimulated for 24 hours until sample collection) was clarified by mixing with trichloroacetic acid (200 μl, 30%

by weight; Sigma Aldrich, St. Louis, MO) by vortex, followed by centrifugation at 8,000xg for 5 minutes. An equal volume of Ehrlich reagent (2% p-dimethylaminobenzaldehyde in glacial acetic acid) was added to the clarified supernatant, and optical density at 490 nm was measured.

Supplementary Method M3. Proteomics Analysis.

Cells were harvested when reached 80% confluency and hMSC pellets were resuspended in protein extracting buffer containing protease inhibitor. The samples were ultra-sonicated for 2 min on ice and the extracted protein concentration was determined by Bradford assay (Bio-Rad, Hercules, CA). The proteins were then digested by modified Filter Aided Sample Prep (FASP) method [2]. Briefly, 100 µg protein was vacuum-dried and resuspended in 8 M urea solution to a final volume of 200 µL, then 10 mM dithiothreitol (DTT) and 50 mM iodoacetamide (IAA) were added for reduction and alkylation respectively. Samples were transferred to a 10 kDa filter and centrifuged with 14000 g for 30 min. After washing with 200 µL of 8 M urea and 200 µL of ammonium bicarbonate, the extracts were centrifuged at 14000 g for 30 min. Then 2 µg trypsin was added for digestion at 37°C overnight. After that, peptides were collected and vacuum-dried.

An externally calibrated Thermo Q Exactive HF (high-resolution electrospray tandem mass spectrometer, MS, Thermo Scientific) was used in conjunction with Dionex UltiMate3000 RSLCnano System. The solution of 1 µg peptides in 0.1% formic acid was injected into a 50 µL loop and loaded onto the trap column (Thermo µ-Precolumn 5 mm, with nanoViper tubing 30 µm i.d. × 10 cm). The flow rate was set to 300 nL/min for separation on the analytical column (Acclaim pepmap RSLC 75 µM× 15 cm nanoviper). Mobile phase A was composed of 99.9% H2O (EMD Omni Solvent) with 0.1% formic acid and mobile phase B was composed of 99.9% acetonitrile with 0.1% formic acid. A 120 min-stepped gradient from 3% to 45% of phase B was performed. The LC eluent was directly nano-sprayed into Q Exactive HF MS. During the chromatographic separation, the Q Exactive HF was operated in a data-dependent mode and under direct control of the Thermo Excalibur 3.1.66 (Thermo Scientific). The MS data were acquired at 20 data-dependent collisional-induced-dissociation (CID) MS/MS scans per full scan (350 to 1700 m/z). The spray voltage for Thermo Scientific™ LTQ was 2.0 kV and the capillary temperature was set at 200°C. A survey full scan (m/z = 350–1700) and the five most intense ions were selected for a zoom scan to determine the charge state, after which MS/MS was triggered in Pulsed-Q Dissociation mode (PQD) with minimum signal required (1000), isolation width 2.0, normalized collision energy 27.0. All measurements were performed at room temperature. Raw files were analyzed by Maxquant 1.6 followed protein identification and relative comparison in Scaffold 4.4. Gene ontology (GO) annotation was carried out by WebGestalt while canonical pathway, diseases and functions analysis was performed by Ingenuity Pathway Analysis (IPA) (Qiagen).

Supplementary Method M4. Western Blot Assay.
hMSCs on culture plate were washed with PBS, and directly lysed in radio-immunoprecipitation assay (RIPA) buffer (150 mM sodium chloride, 1.0% Trition X-100, 0.5% sodium deoxycholate, 0.1% sodium dodecyl sulfate, 50 mM Tris, pH 8, 2 µg/mL Aprotinin, 5 µg/mL Leupeptin, 5 µg/mL Antipain, 1 mM PMSF protease inhibitor), and homogenized by sonification using a Sonic Dismembrator 100 (Fisher Scientific, Hampton, NJ). Samples were then digested for 20 min on ice, and centrifuged at 14,000 rpm for 20 min at 4°C. The supernatant was collected, and a Bradford assay was performed to determine the protein concentration. Protein lysate concentration was normalized, and 20 µg of each sample was denatured at 95°C in 2 x Laemmli Sample buffer.

Proteins were separated by 15% BIS-Tris-SDS gels and transferred onto a nitrocellulose membrane (Bio-rad, Hercules, CA). For the detection of non-phosphorylated proteins, the membranes were blocked for 30 min in 3% skim milk (w/v) in Tris-buffered saline (10mM Tris-HCl, pH 7.5, and 150mM NaCl) with 0.1% Tween 20 (v/v) (TBST), or in 3% bovine serum albumin in TBST. Membranes were incubated overnight in the presence of the primary antibody diluted (1:1000) in the corresponding blocking buffer at 4°C. Afterward, the membranes were washed four times for 10 min each with TBST and then incubated with an IR secondary (LI-COR, Lincoln, NE) at 1:10,000 for 180 min at room temperature. Blots were then washed another four times for 10 min each with TBST and processed using the LI-COR OdysseyCLx (LI-COR, Lincoln, NE). Images were analyzed using ImageJ software for band density, and the band density of proteins of interest was normalized to the band density of endogenous control β-actin.

Supplementary Figures

Supplementary Figure S1. Immuno-response of culture-expanded human mesenchymal stem cells (hMSCs) depends on passage number. (A) The IDO activity and (B) mRNA expression of Nk-kB and COX2 determined by RT-PCR. ** indicates p<0.01.

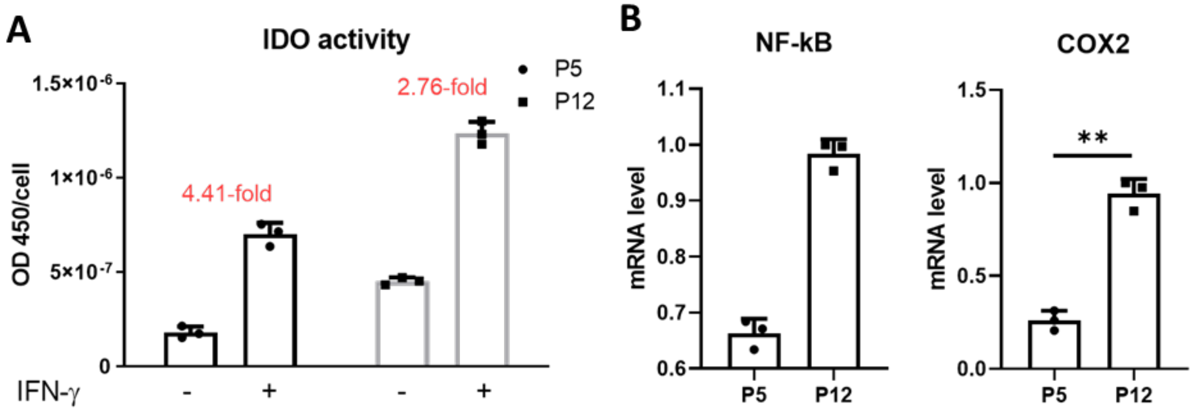

Supplementary Figure S2. Immunomodulation of human mesenchymal stem cells (hMSCs) at different passages. (A) Anti-inflammation factors (HGF, IL-10, and CXCL10). (B) Pro-inflammation factors (TNF-α, IL1-β, and IL6); Determined by enzyme-linked immunosorbent assay (ELISA) normalized to cell number. ** indicates $p<0.01$. ND: not detectable. NS: not statistically significant.

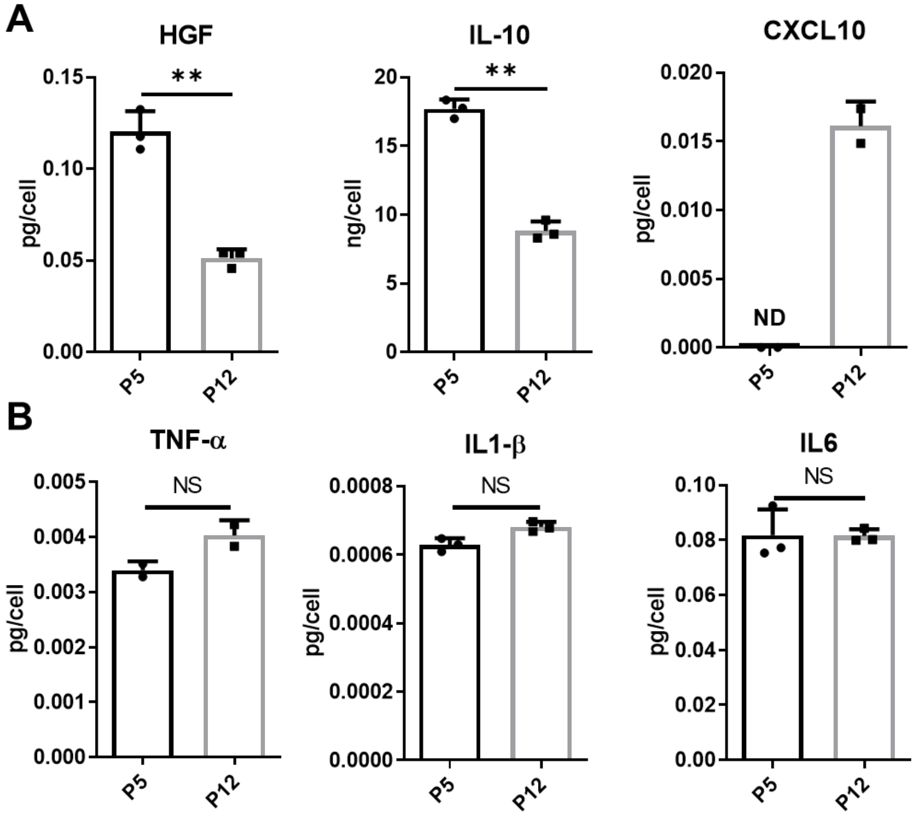

Supplementary Figure S3. OCR and ECAR in hMSCs at P5 and P12, determined by seahorse flux analyzer. (A) OCR measurement of hMSCs at baseline and after stressed by oligomycin/FCCP. (B) ECAR measurement of hMSCs at baseline and after stressed by oligomycin/FCCP.

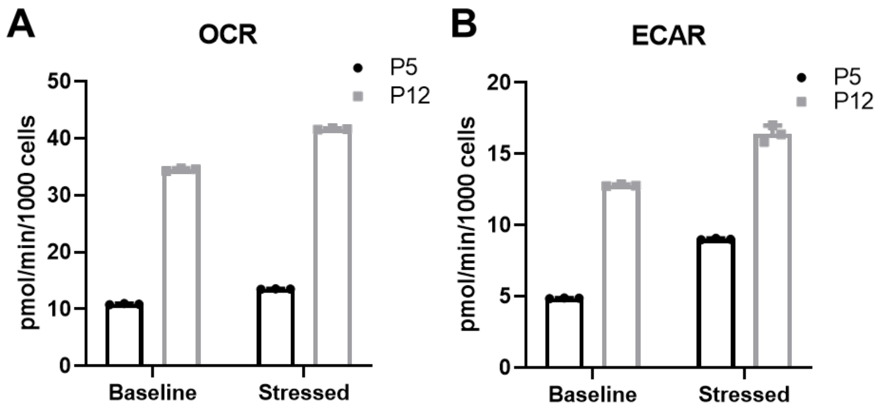

Supplementary Figure S4. Quantitative analysis of proteomics for human mesenchymal stem cells (hMSCs) at P4, P8, and P12. (A) Venn diagram shows the distribution of proteins identified in each passage of hMSCs. There are 587 proteins in common across three experimental groups. (B) Principal component analysis (PCA) demonstrated the separation of hMSCs at P4, P8, and P12 as categorized with the protein identified in proteomics. (C) Volcano plots with yellow dots indicated the proteins are more than 10-fold change or proteins associated with NAD metabolism (e.g. HADHA, NNMT, HSD17B4, OGDH and DLD).

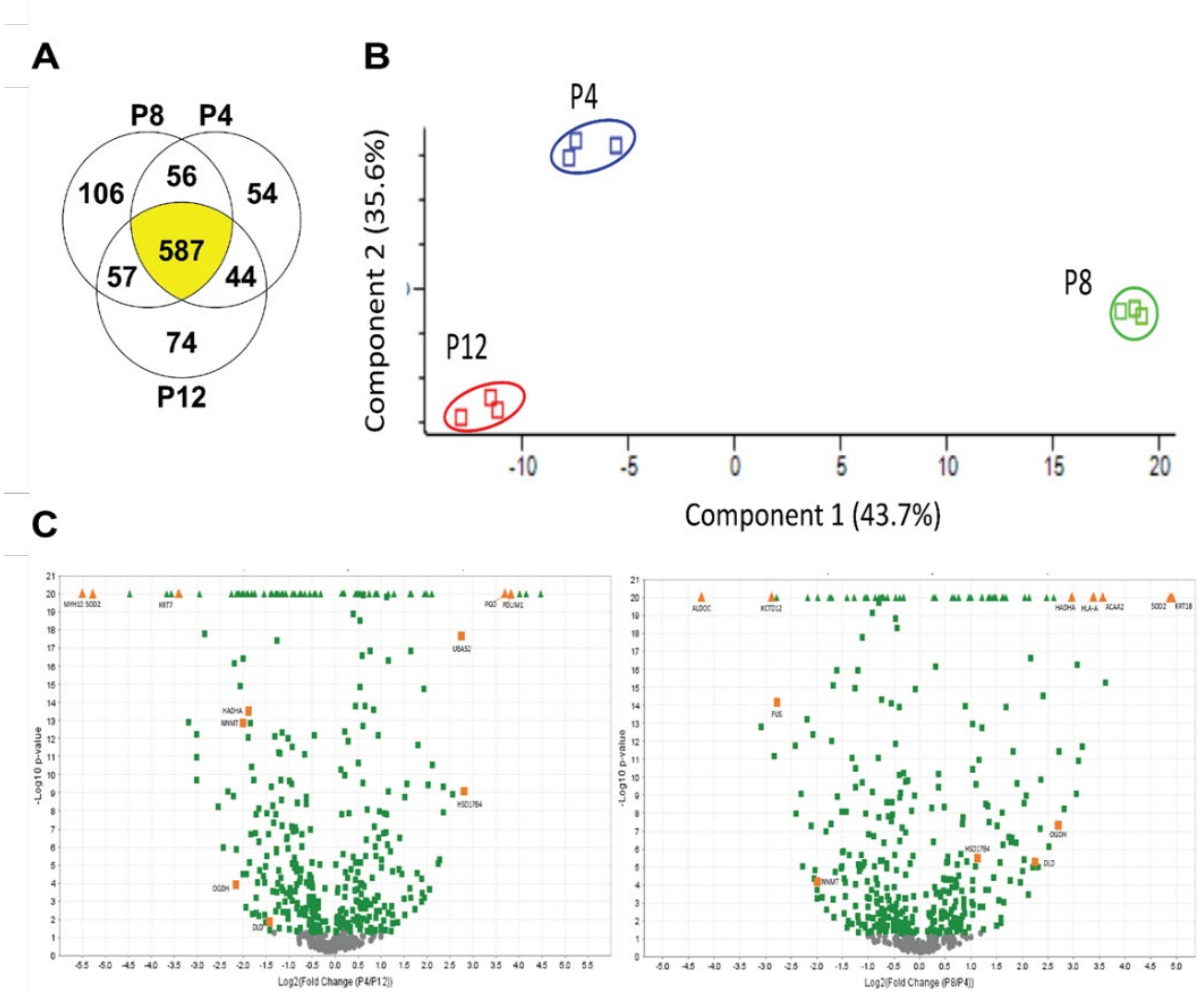

Supplementary Figure S5. Gene oncology and pathway investigation of differentially expressed proteins (DEPs) between early passage and late passage human mesenchymal stem cells (hMSCs). (A) Pie chart of gene ontology (GO) analysis for proteins in biological process, cellular component, and molecular function. (B) Heat map shows the differentially expressed proteins between hMSCs of low passage (P4) and MSCs of high passages (P8, P12).

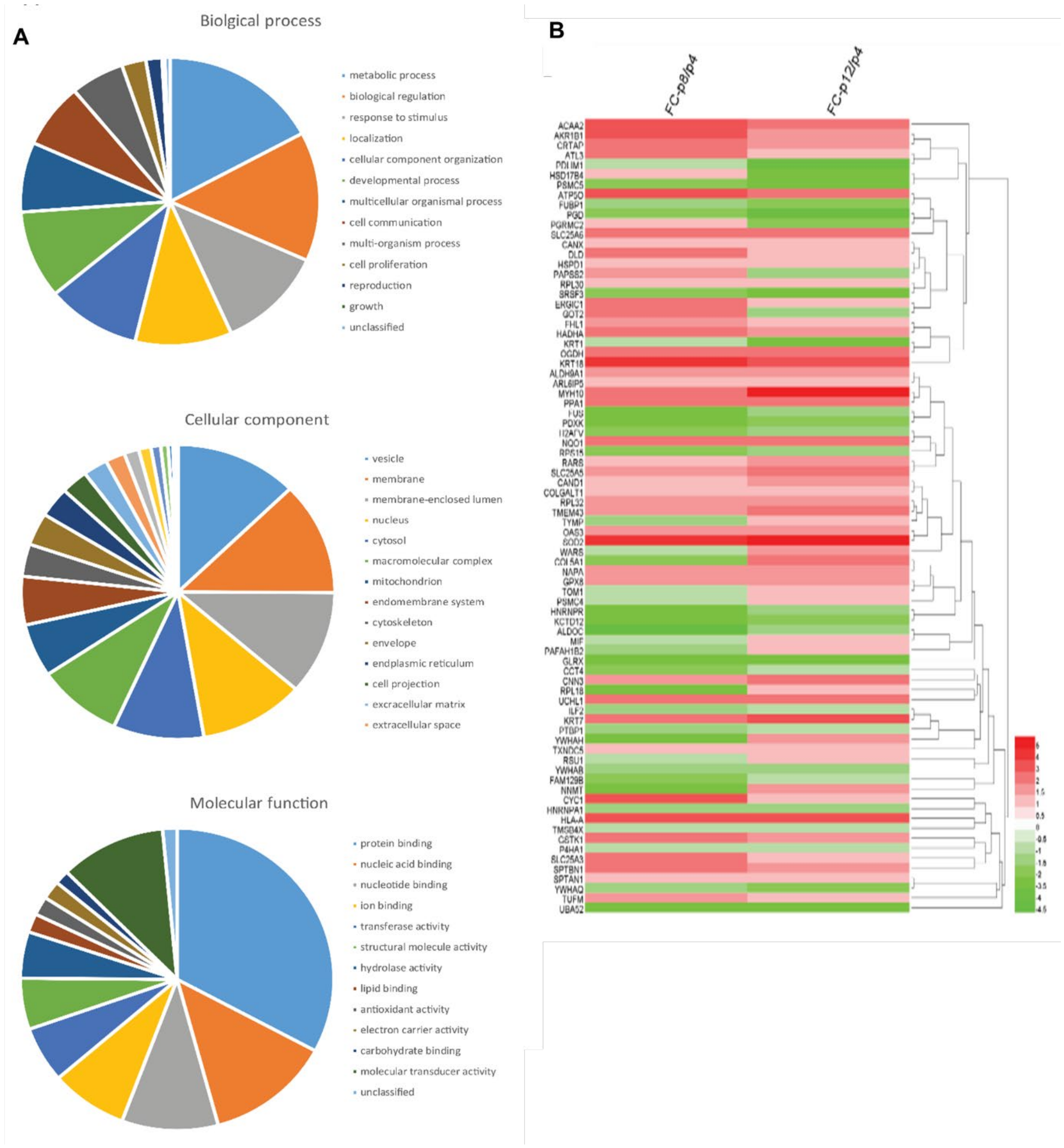

Supplementary Figure S6. Kyoto Encyclopedia of Genes and Genomes (KEGG) pathway map of glycolysis and gluconeogenesis. Mappable enzymes within the pathway are highlighted in green and were identified with the web based tool: http://www.webgestalt.org/. Up-regulated enzymes in P12 compared to P4 hMSCs are labeled in red color.

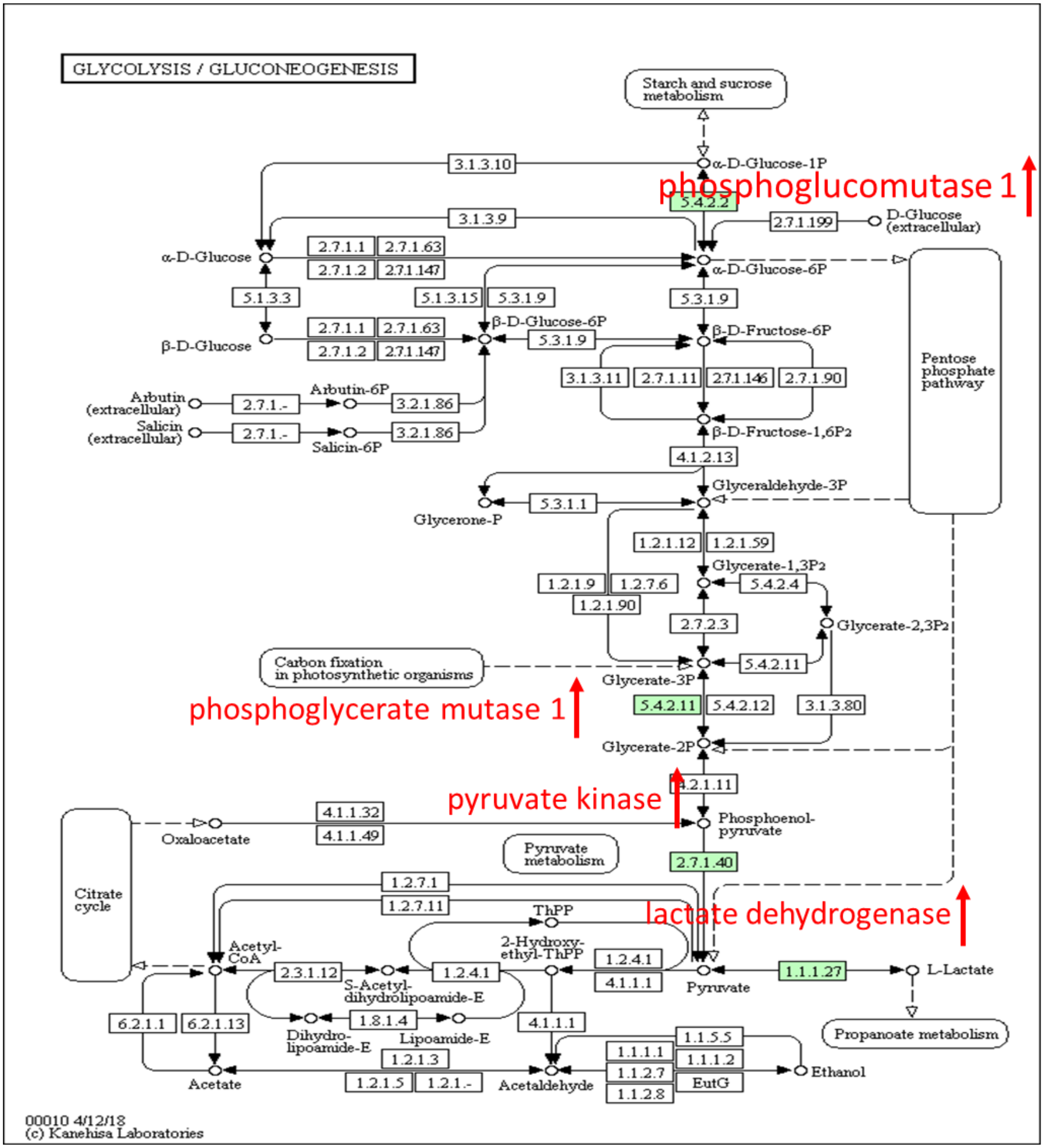

Supplementary Figure S7. KEGG pathway map of pyruvate metabolism. Mappable enzymes within the pathway are highlighted in green and were identified with the web based tool: http://www.webgestalt.org/. Up-regulated enzymes in P12 compared to P4 hMSCs are labeled in red color.

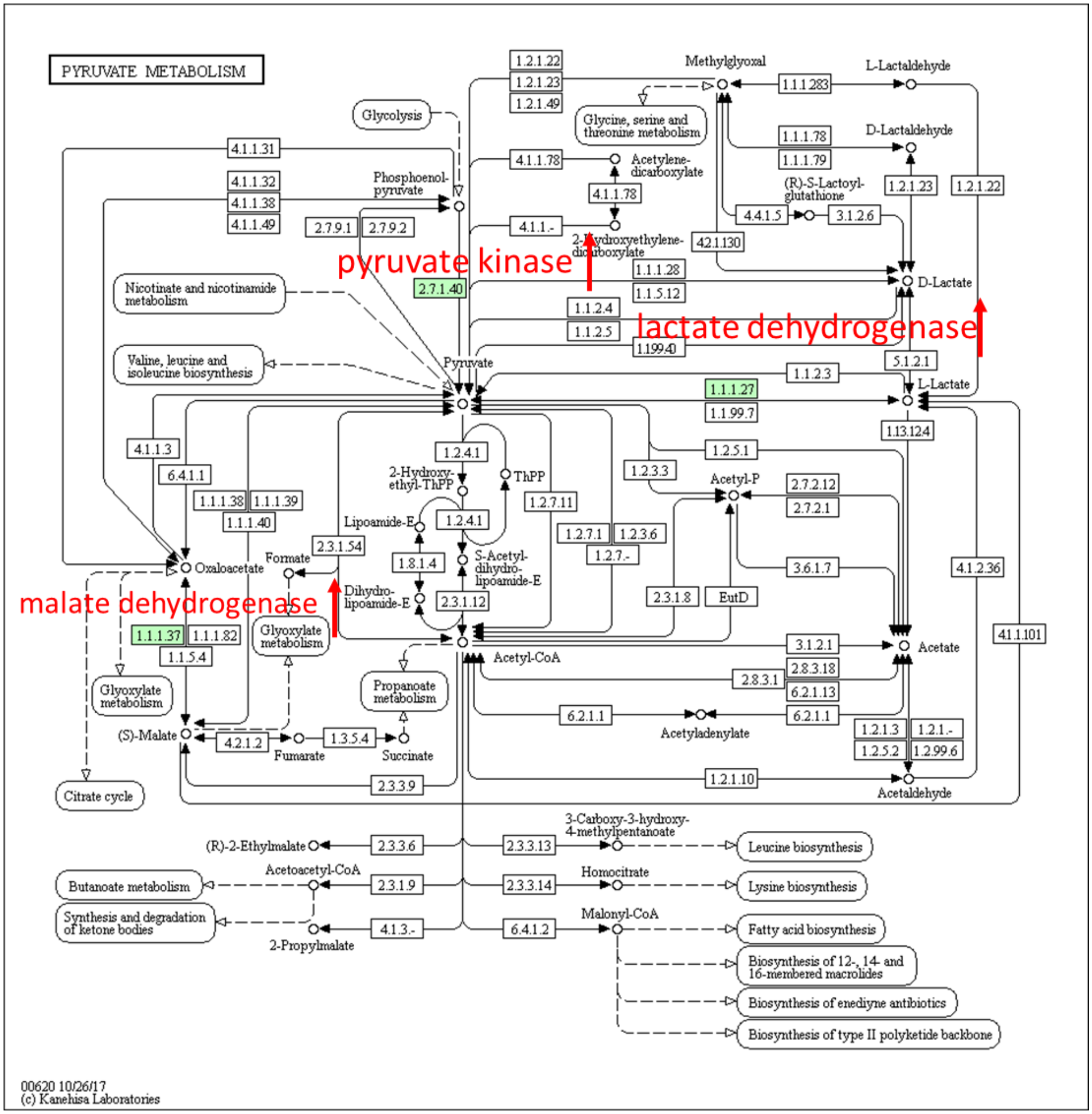

Supplementary Figure S8. KEGG pathway map of carbon metabolism. Mappable enzymes within the pathway are highlighted in green and were identified with the web based tool: http://www.webgestalt.org/. Up-regulated enzymes in P12 compared to P4 hMSCs are labeled in red color.

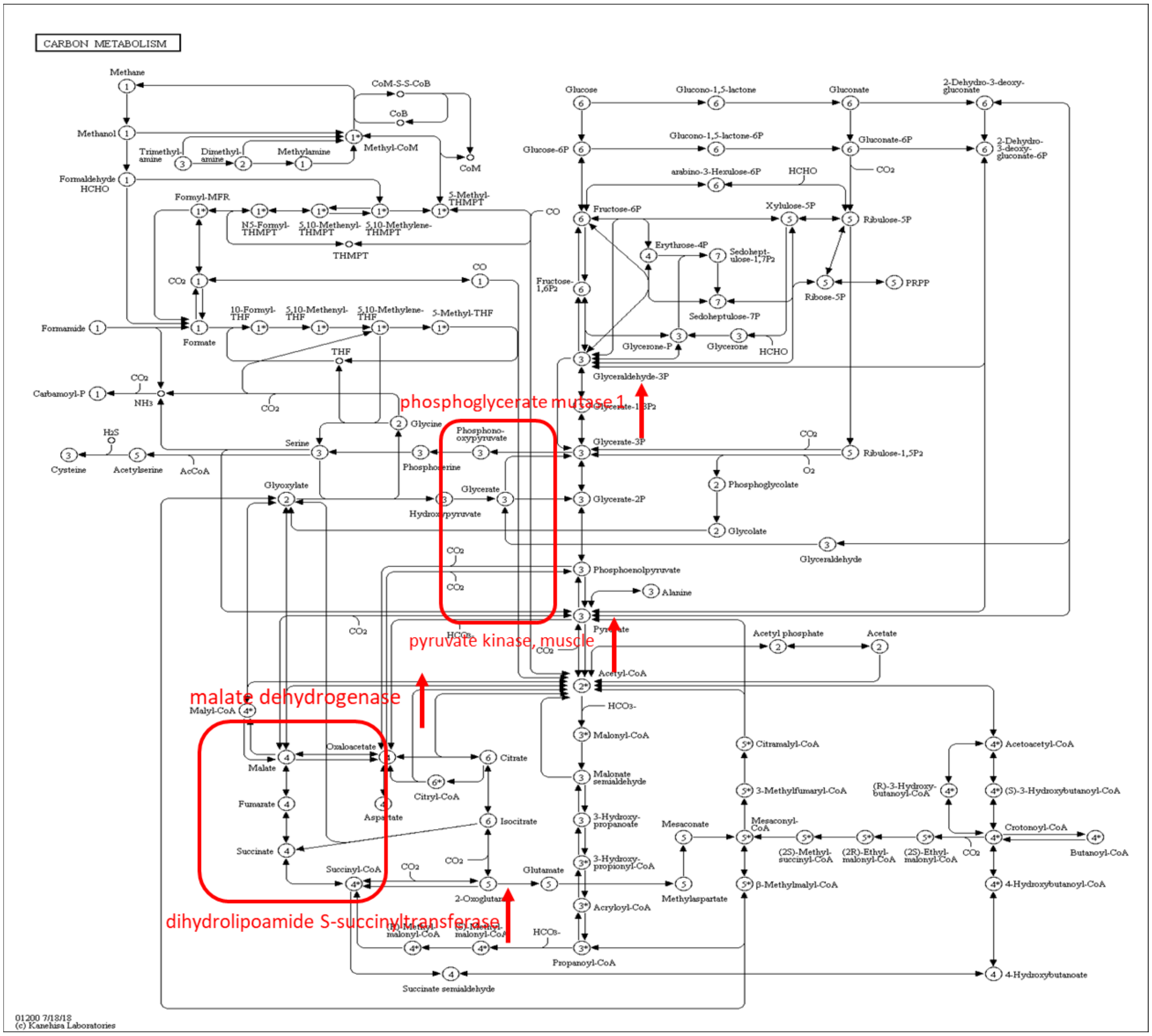

Supplementary Figure S9. Ingenuity Pathway Analysis (IPA) of early passage and late passage of hMSCs. (A) Canonical pathways are identified by enrichment of different expressed proteins (DEPs). Y-axis is the identified pathways (top 5). The x-axis corresponds to the –log of the P-value (Fisher's exact test) and the orange points on each pathway bar represent the ratio of the number of genes in a given pathway that meet the cutoff criteria, divided by the total number of genes that map to that pathway. (B) DEPs between P12 and P4 hMSCs enriched in the disease and disorder category from IPAs (top 5).

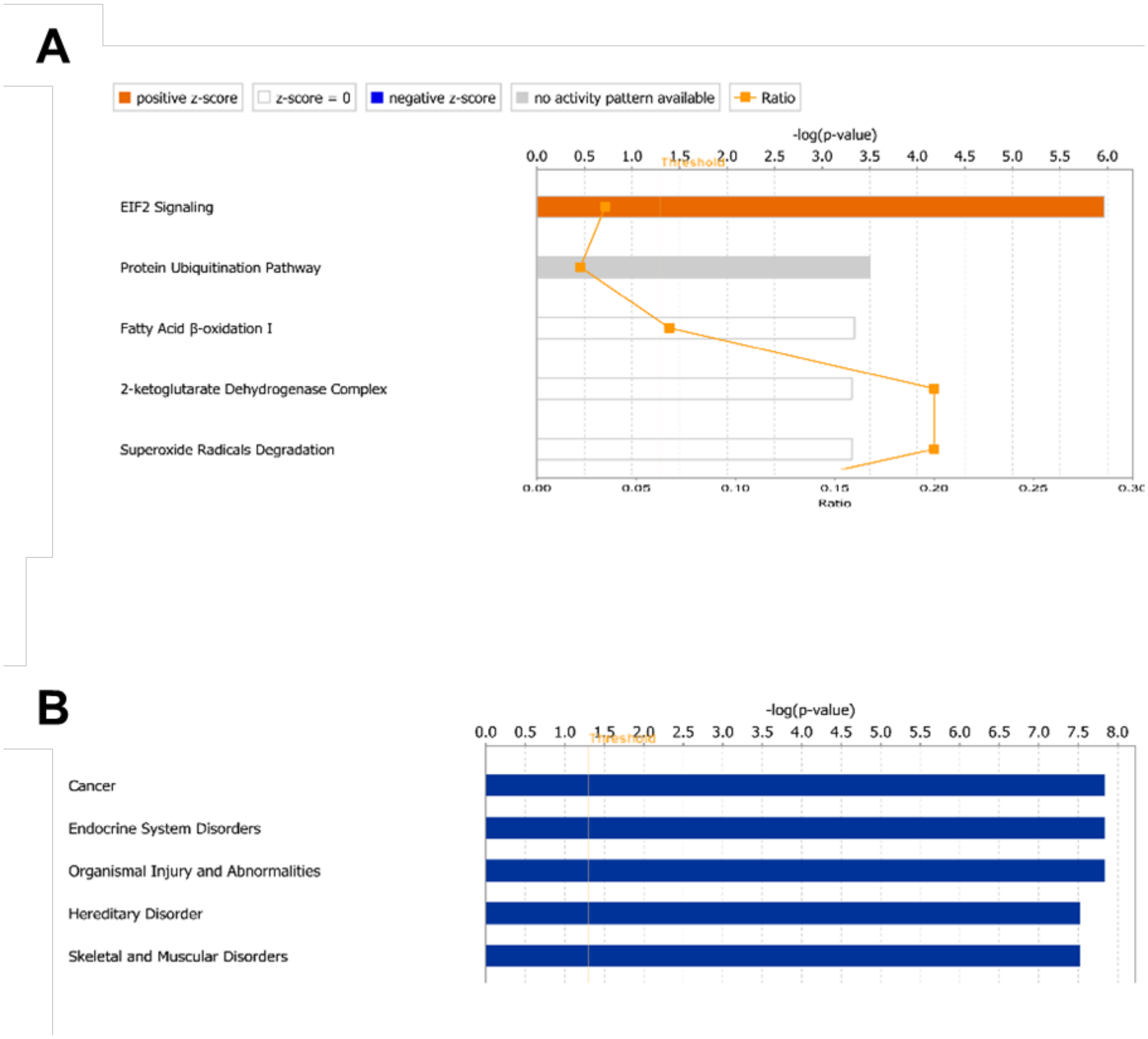

Supplementary Figure S10. Proteomics analysis reveals the potential pathways related to NAD$^+$/NADH redox cycle: Fatty acid β-oxidation. (A) Differential expression of proteins that are encoded with genes related to fatty acid β-oxidation pathway (P12 over P4). (B) Differential protein expression is denoted as pink (upregulated) and green (downregulated) in the Fatty acid β-oxidation canonical pathway. (C) Simplified illustration of involvement of NAD$^+$/NADH in fatty acid β-oxidation pathway.

A

Entrez Gene Name	Gene	Expr Fold Change	Location	Type(s)
acetyl-CoA acyltransferase 2	ACAA2	7.6	Cytoplasm	enzyme
hydroxyacyl-CoA dehydrogenase trifunctional multienzyme complex subunit alpha	HADHA	3.5	Cytoplasm	enzyme
hydroxysteroid 17-beta dehydrogenase 4	HSD17B4	-10	Cytoplasm	enzyme

B

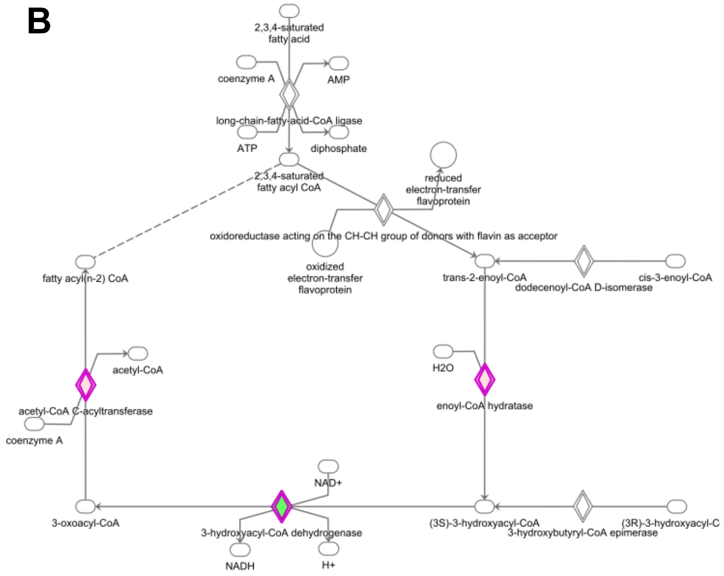

C

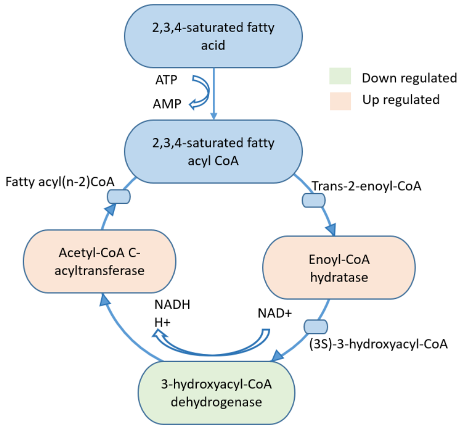

Supplementary Figure S11. Proteomics analysis reveals the pathways related to NAD+/NADH redox cycle: 2-ketoglutarate dehydrogenase complex pathway. (A) Differential expression of proteins that are encoded with genes related to 2-ketoglutarate dehydrogenase complex pathway (P12 over P4). (B) Differential protein expression is denoted as pink (upregulated) in the 2-ketoglutarate dehydrogenase complex canonical pathway. (C) Simplified illustration of involvement of NAD+/NADH in 2-ketoglutarate dehydrogenase complex pathway.

A

Entrez Gene Name	Gene	Expr Fold Change	Expected	Location	Type(s)
dihydrolipoamide dehydrogenase	DLD	2.6	Up	Cytoplasm	enzyme
oxoglutarate dehydrogenase	OGDH	4.2	Up	Cytoplasm	enzyme

B

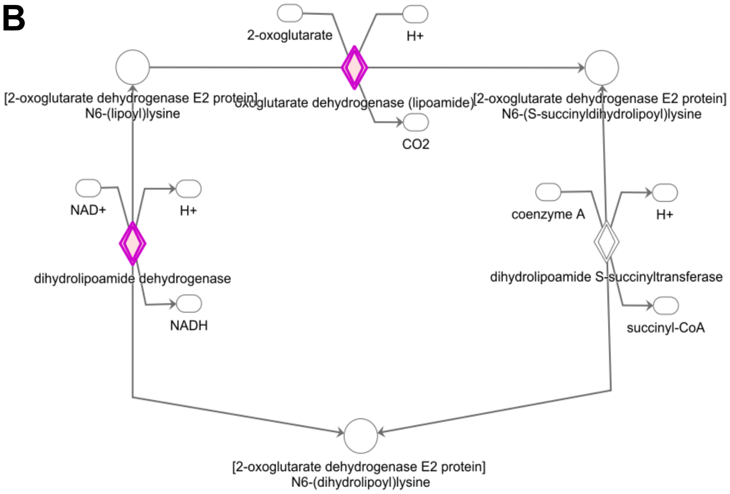

C

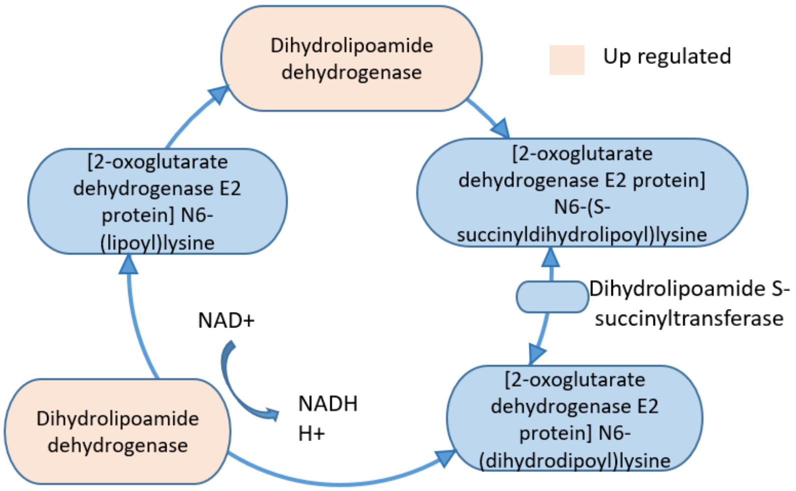

Supplementary Figure S12. Proteomics analysis reveals the pathways pathways related to NAD$^+$/NADH redox cycle: superoxide radicals degradation. (A) Differential expression of proteins that are encoded with genes related to superoxide radicals degradation pathway (P12 over P4). (B) Differential protein expression is denoted as red (upregulated) and green (downregulated) in the superoxide radicals degradation canonical pathway.

A

Entrez Gene Name	Gene Symbol	Expr Fold Change	Expected	Location	Type(s)
NAD(P)H quinone dehydrogenase 1	NQO1	7.6	Up	Cytoplasm	enzyme
superoxide dismutase 2	SOD2	37	Up	Cytoplasm	enzyme

B

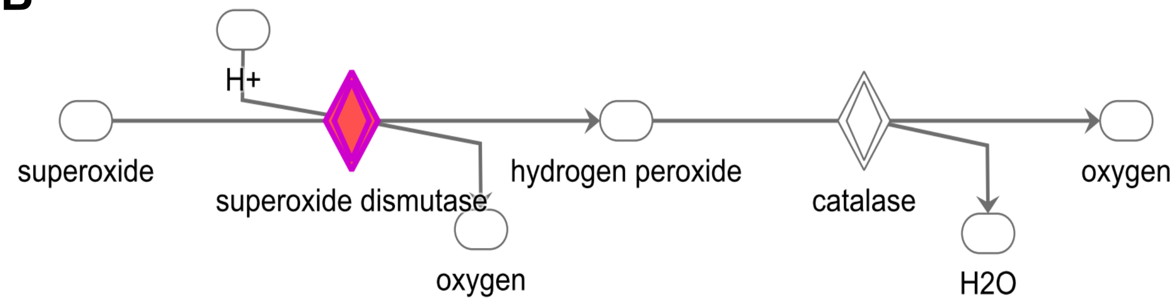

Supplementary Figure S13. Proteomics analysis reveals five differentially expressed proteins (DEPs) participate in the NAD metabolism process. (A) Differential expressed proteins (DEPs) for P12 over P4 hMSCs associated with metabolism and NAD^{+}/NADH redox cycle identified via IPA and (B) Protein-protein interaction of these five DEPs. Illustration of labeling in the protein-protein interaction is from IPA.

A

Protein name	Uniprot ID	Gene name	FC (p12/p4)
Trifunctional enzyme subunit alpha, mitochondrial	ECHA_HUMAN	HADHA	3.5
Nicotinamide N-methyltransferase	NNMT_HUMAN	NNMT	3.7
Peroxisomal multifunctional enzyme type 2	DHB4_HUMAN	HSD17B4	0.1
2-oxoglutarate dehydrogenase, mitochondrial	ODO1_HUMAN	OGDH	4.2
Dihydrolipoyl dehydrogenase, mitochondrial	DLDH_HUMAN	DLD	2.6

B

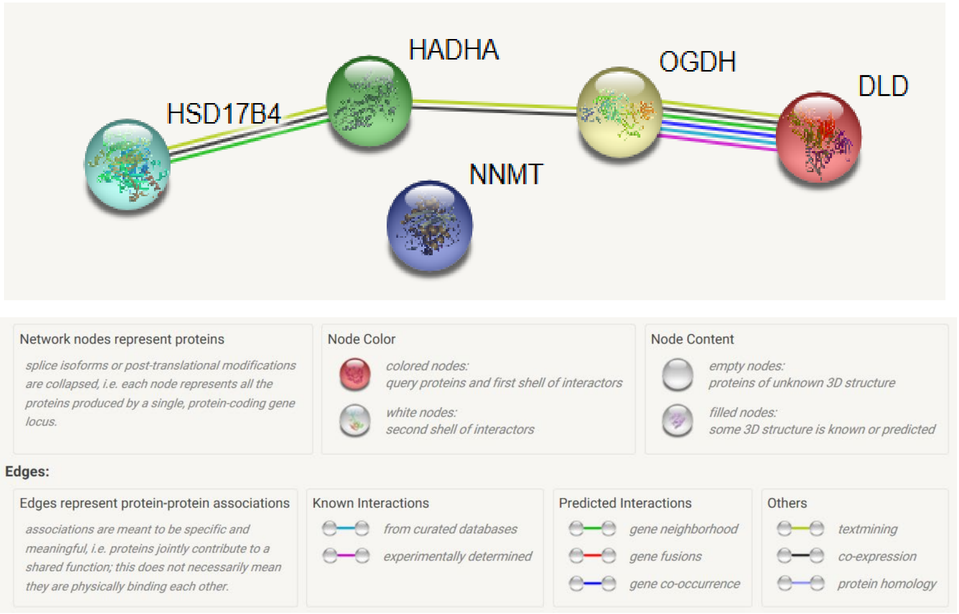

Supplementary Figure S14. Intracellular NAD^+ level and NAD^+/NADH ratio measured in alternative donors and passages of hMSCs. (A) and (B) NAD^+ and NADH level and NAD^+/NADH ratio of donor 2 hMSCs at P4, P7, and P9. (C) and (D) NAD^+ and NADH level and NAD^+/NADH ratio of donor 3 hMSCs at P4, P5, P6, P9, P10, P11, and P12.

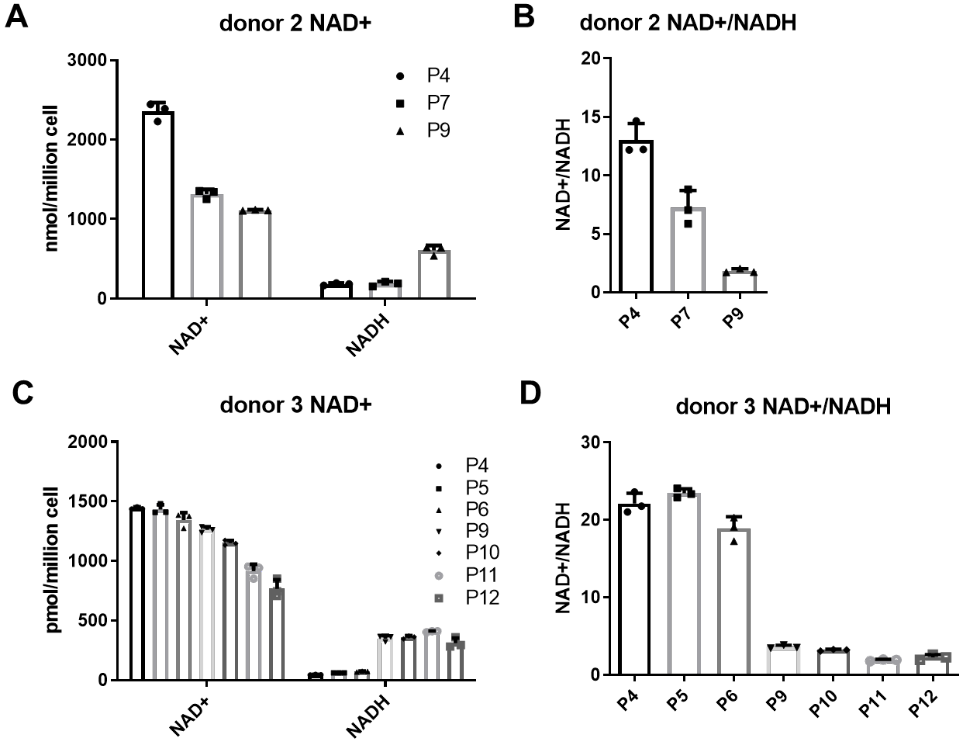

Supplementary Figure S15. Proposed mechanism of regulatory role of NAD^+/NADH redox cycle in hMSCs cellular homeostasis during *in vitro* culture expansion. Culture expansion of hMSCs results in accumulation of DNA damage, which further activates PARP signal and causes the intracellular NAD^+ decrease. Imbalanced NAD^+/NADH level causes NAD^+ dependent Sirtuin inactivation, which down-regulates several pathways, including mitochondrial biogenesis, anti-oxydant protection, immunomodulation. Dysfunction of mitochondria further accumulates NADH and consumes NAD^+ to maintain cellular function, leading to energy metabolism shift from glycolysis towards OXPHOS. Maintaining intracellular NAD^+ pool size via supplement of NAD^+ precursors could enhance hMSCs resistance to senescence during replicative expansion.

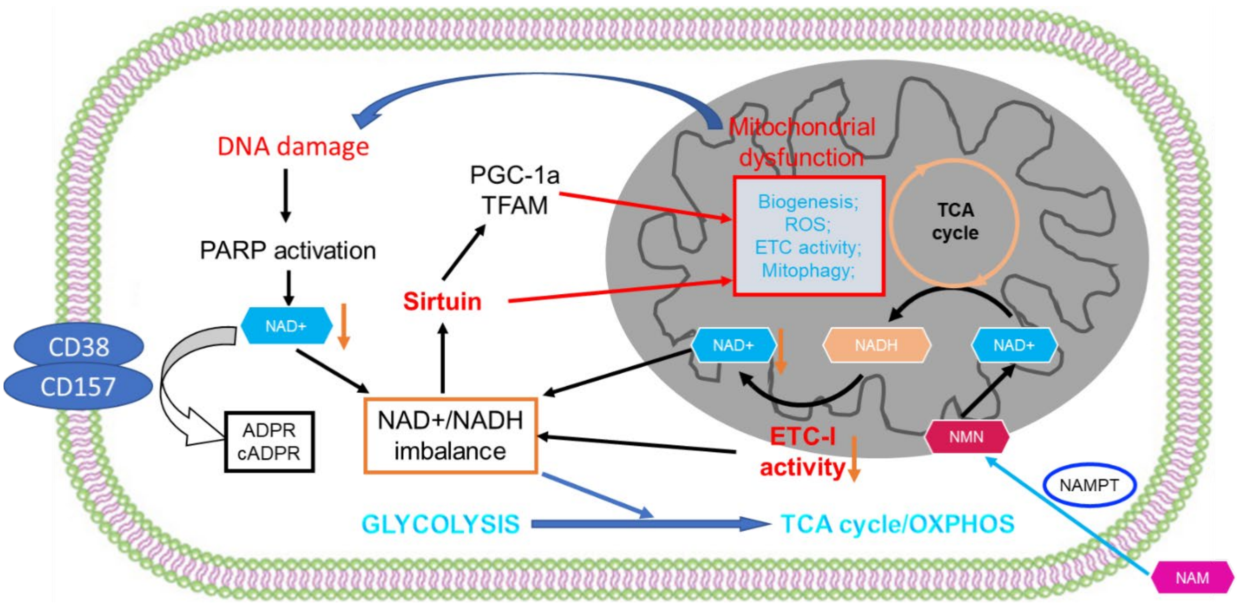

Supplementary Figure S16. Nuclear magnetic resonance (NMR) spectrum of human mesenchymal stem cell (hMSC) culture medium, NAD$^+$, NADH, and NAD$^+$ precursor NAM. NMR spectra of different samples were obtained on a Bruker 500M spectrometer at 200 MHz. The spectrum was taken in deuterated chloroform at 20ºC. The peaks for fresh culture medium (green), NAD$^+$ (yellow), NADH (red), and NAM (black) were labeled in the spectra.

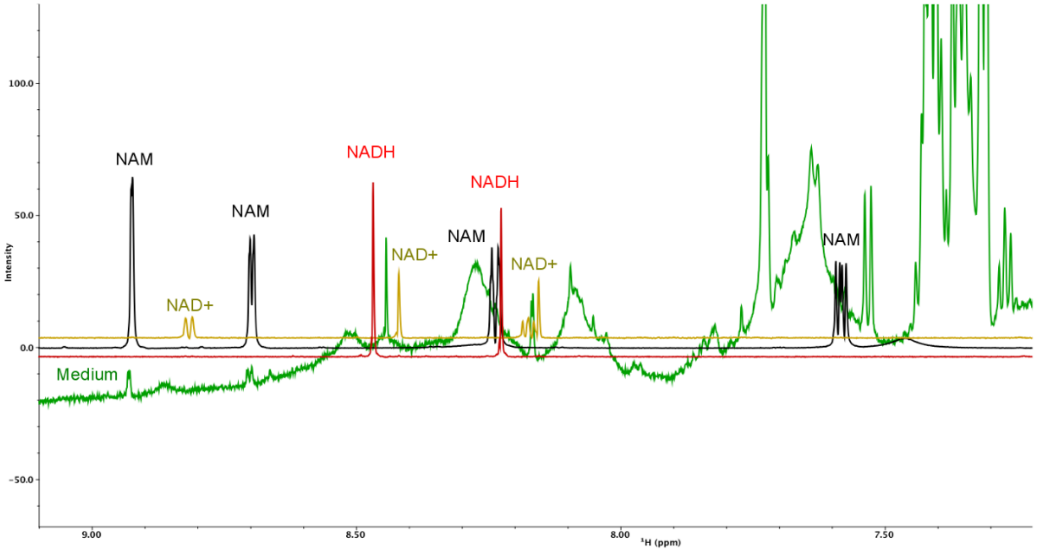

Supplementary Figure S17. Cell cycle analysis of senescent hMSCs wth NAM treatment. (A) hMSCs at P12. (B) hMSCs at P12 with 96 hr treatment of NAM.

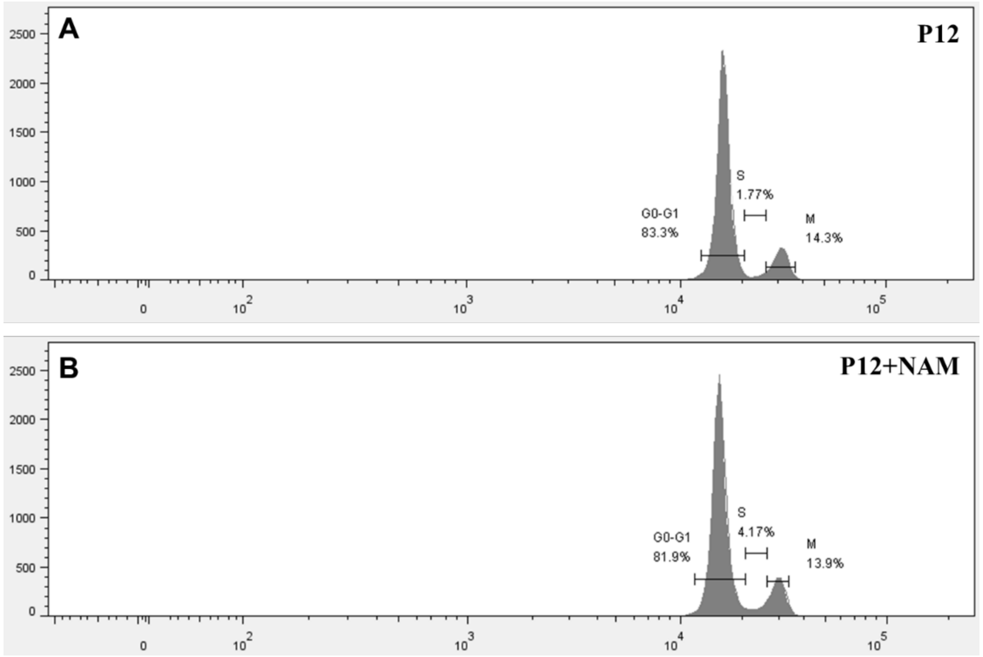

Supplementary Figure S18. Lineage-specific differentiation of hMSCs at P5 and P12. mRNA expression of lineage-specific marker expression by RT-PCR. (A) Relative mRNA expression for osteogenic differentiation of hMSCs at P5 and P12; (B) Relative mRNA expression for adipogenic differentiation of hMSCs at P5 and P12. (C) Representative images for Oil red O staining and Von Kossa staining. Scale bar: 100 µm. * indicates p<0.05; **, p<0.01; ***, p<0.001.

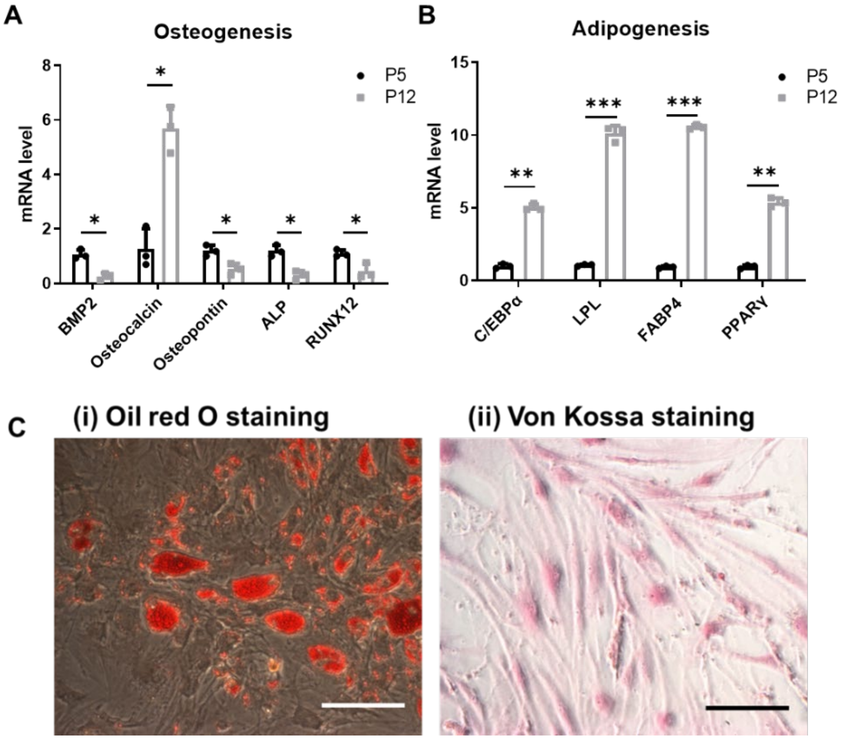

Supplementary Figure S19. Example of gating strategy for flow cytometry analysis in hMSCs at early and late passage.

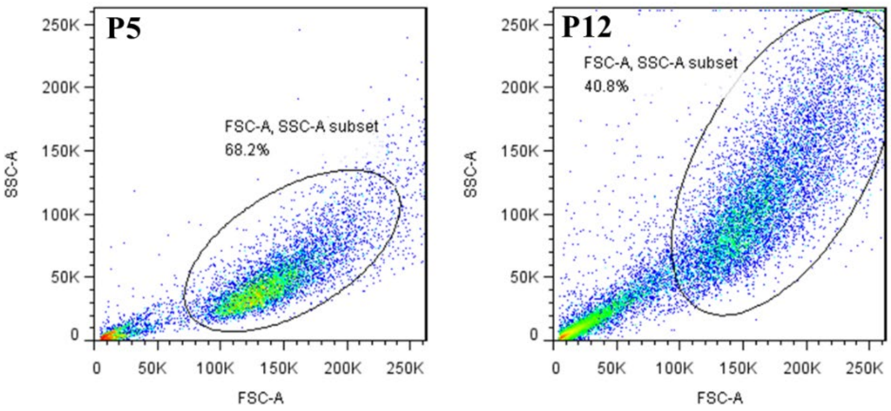

Supplementary Tables

Supplementary Table S1. KEGG identified key pathways that are altered during culture expansion of hMSCs.

ID	Name	#Gene	FDR
hsa01100	Metabolic pathways - Homo sapiens (human)	12	1.75e-04
hsa00010	Glycolysis / Gluconeogenesis - Homo sapiens (human)	5	1.85e-02
hsa00270	Cysteine and methionine metabolism - Homo sapiens (human)	5	1.85e-02
hsa00620	Pyruvate metabolism - Homo sapiens (human)	5	1.85e-02
hsa01200	Carbon metabolism - Homo sapiens (human)	5	1.85e-02
hsa04145	Phagosome - Homo sapiens (human)	5	1.85e-02
hsa04151	PI3K-Akt signaling pathway - Homo sapiens (human)	5	1.85e-02
hsa04510	Focal adhesion - Homo sapiens (human)	5	1.85e-02
hsa04612	Antigen processing and presentation - Homo sapiens (human)	5	1.85e-02

Supplementary Table S2. A list of antibodies.

Primary Antibody	Origin/ Isotype	Supplier/Cat#	Dilution
Sirt-1	mouse monoclonal IgG1	SANTACRUZ/sc-74465 FITC	Flow cytometry: 1:200
Sirt-3	mouse monoclonal IgG2b	SANTACRUZ/sc-365175	Flow cytometry: 1:200
CD38	mouse monoclonal IgG1	SANTACRUZ/sc-374650	Flow cytometry: 1:200
CD73	mouse monoclonal IgG1	SANTACRUZ/sc-398260	Flow cytometry: 1:200
NAMPT	mouse monoclonal IgG2b	SANTACRUZ/sc-393444 FITC	Flow cytometry: 1:200
PGC-1α	Rabbit IgG	Cell Signaling/2178S	Western blot: 1:1000
Sirt-1	Rabbit IgG	Cell Signaling/2310S	Western blot: 1:1000
Sirt-3	Rabbit IgG	Cell Signaling/2627S	Western blot: 1:1000
CD38	Rabbit IgG	Cell Signaling/14637S	Western blot: 1:1000
CD73	Rabbit IgG	Cell Signaling/13160S	Western blot: 1:1000
NAMPT	Rabbit IgG	Cell Signaling/86634S	Western blot: 1:1000
**Secondary Antibody**	**Origin/ Isotype**	**Supplier/ Cat#**	**Dilution**
AF647	Goat anti-Mouse IgG (H+L)	Invitrogen/A-21235	Flow cytometry: 1:500
AF488	Goat anti-Mouse IgG1	Invitrogen/ A-21121	Flow cytometry: 1:500
PE	Goat anti-Mouse IgG1	Invitrogen/ P-21129	Flow cytometry: 1:500
IRDye® 800CW	Goat anti-Rabbit IgG (H + L)	LI-COR/ 926-32211	Western blot: 1:10,000

Supplementary Table S3. Primer sequences of target genes in RT-PCR analysis.

Gene	Forward primer 5' to 3'	Reverse primer 5' to 3'
Oct-4	CAGCAGATCAGCCACATCGCC	TGAGAAAGGAGACCCAGCAGCC
Nanog	CCTGTGATTTGTGGGCCTG	GACAGTCTCCGTGTGAGGCAT
SOX2	GTATCAGGAGTTGTCAAGGCAGAG	TCCTAGTCTTAAAGAGGCAGCAAAC
P53	CTGGACGACAGGCAGACTTT	GCACAAACACGAACCTCAAA
P15 (CDKN2B)	GCTGTTTCATCAGCAGCCTAA	TTCCACAATGGAGCTAGAAGCA
P21	GAGACTCTCAGGGTCGAAAACG	GGATTAGGGCTTCCTCTTGGAG
TFEB	CCTGGTGGAGATTCCCTGTCT	CAGGACCAGTTGCCTCAGATG
BECN1	ACTGTGTTGCTGCTCCATGCT	AACGGCAGCTCCTTAGATTTGT
LAMP1	TCACACGTAGGACGCATGAAG	GAAGCGCTCCAGACACTCATC
MFN1	AAATGCTCAAAGGGTGCTCCT	GATGCATTATCTGGCGTTGCT
MFN2	GATGCCTGTCACCAAGGTGTT	TGCTTTTTGGGAGAGGTGTTG
FIS1	CTGGTGCGGAGCAAGTACAAT	CACGGCCAGGTAGAAGACGTA
DNM1L	CGATGCACTTTTCTCCAGCAC	TTCCGTTGTTTCTTGCCTCTG
NRF1	AGAAGGGACACCTGGCCATAG	AGGTTGCATCAAGGAACATGG
NRF2	ACAATTTGGGCACTGTGGTTT	CCGTAAGAGGATGGTGCAAAA
NDUFS8	CGCTATGACATCGACATGACC	CCGTGGAGAACTCAAAGTTGG
SDHB	AGTGCATTCTCTGTGCCTGCT	CAGCGATAGGCCTGCATAAGA
UQCRC1	GTGCTGTGGCCAACAAGCTAT	TCGGTCACAGACAAAGTGTGC
COX5B	TTGGAGAGGGAGATCATGCTG	TTGTTGGAGATGGAGGGGACT
ATP5F1B	CTGGCCACTGACATGGGTACT	GTCAAGTCATCAGCAGGCACA
PDK1	AAACAGGGGAGCTTTGTCTGG	CTGCCCATTCACATCCCTCTA
HK2	TGGTGTAGCTCCTCTGCTGCT	TGTGGGCACCCTTTAGTGAAC
PKM2	AAAAATGGATGCCCAGAGGAC	GAGTCGGCTTCAATGGAACAA
LDHA	CCTTGAGCCAGGTGGATGTTT	CACTGGATCCCAGGATGTGAC
G6PD (GL6PD)	CTACCCGAGCCCAGCTACATT	TTCTGTTGGGCTGGAGTGAGT
6PGD (6PGLD)	CCATGCCCTGTTTTACCACTG	AGGTGTGAGCCCCGAAGTAAT
TALDO1	CTGTCATCAACCTGGGAAGGA	GGGCGAAGGAGAAGAGTAACG
TKTL1	ACCTTGGGATTCTGTGTGCTG	CCTAACAAGCTTTCGCTGCTG

SIRT1	TCGCAACTATACCCAGAACATAGACA	CTGTTGCAAAGGAACCATGACA
SIRT3	GCATCCCTGCCTCAAAGC	CGTCAGCCCGAATGTCCTC
PARP1	TCTCAGGGAGACCCAATAGGC	GCCCTTGGGTAACTTGCTGAT
PGC1a	TGTGGGTAGCCCATCAAAATG	GCTGAAATTTGCCTCCAGTGA
TFAM	TTTTCCCTGTGGGAGCTTCTC	CAGACTGCTCTGGCCTCAAAC
FOXO1	AGCCTCCCGGGTATGTAACTG	GTAATGGCACGGGAGGAAAGT
FOXO3	GCCACCCTTGGCCTCTAAATA	GAGCGCATGTCATGTTTTCAA
CD38	TTCCCGCAGTTTTTCTTTGAA	GCACCTCTCAGCTGCCTACTG
CD73	GCCTGGGAGCTTACGATTTTG	TAGTGCCCTGGTACTGGTCG
NAMPT	CTCCATTCTGAGCACCAAACG	ATGCCATTTGCTTTTGTCTGG
ALP	CAGTCTGCTGTGCCCCTGC	GTAGTTCTGCTCGTGGACGCC
BMP2	TTCCCCGTGACCAGACTTTTGG	GCCACTTCCACCACGAATCCAT
Osteo-calcin	GGCAGCGAGGTAGTGAAGAGAC	GAAAGCCGATGTGGTCAGCCAA
Osteo-pontin	AGCGGAAAGCCAATGATGAGAGC	ACTTTTGGGGTCTACAACCAGCAT
RUNX2	CCAACCCACGAATGCACTATC	TAGTGAGTGGTGGCGGACATAC
CEBPA	AGCCTTGTTTGTACTGTATG	AAAATGGTGGTTTAGCAGAG
FABP4	ACGAGAGGATGATAAACTGGTGG	GCGAACTTCAGTCCAGGTCAAC
LPL	CTGACCAAGGATAGTGGGATATAG	GGTAACTGAGCGAGACTGTGTCT
PPARγ	AGCCTGCGAAAGCCTTTTGGTG	GGCTTCACATTCAGCAAACCTGG
GAPDH	TCACTGCCACCCAGAAGACTG	GGATGACCTTGCCCACAGC
TUB1a (TUBA1A)	TGAGGAGGTTGGTGTGGATTC	AAAAGCAGCACCTTTGTGACG

References:

1. Rosenberg, J.T. et al. Magnetic resonance contrast and biological effects of intracellular superparamagnetic iron oxides on human mesenchymal stem cells with long-term culture and hypoxic exposure. *Cytotherapy* **15**, 307-322 (2013).
2. Yan, Y. et al. Differential effects of acellular embryonic matrices on pluripotent stem cell expansion and neural differentiation. *Biomaterials* **73**, 231-242 (2015).
